# Anthropometry of Proximal Tibia in Patients Undergoing Total Knee Replacement at a Tertiary Care Hospital

**DOI:** 10.7759/cureus.58758

**Published:** 2024-04-22

**Authors:** Muhammad Amin Chinoy, Naeem Ahmed, Ibrahim Zahid Selod, Tauqeer Khan, Muhammad Waseem Memon, Salman Javed

**Affiliations:** 1 Orthopaedics, The Indus Hospital, Karachi, PAK

**Keywords:** anthropometric variations, osteoarthritis, dimensions, proximal tibia, total knee replacement

## Abstract

Introduction

Total knee arthroplasty is the standard of care treatment for advanced knee osteoarthritis. However, patients frequently continue to have pain and disability after surgery, with one of the most common reasons being a bone-implant mismatch. Notably, there is a significant difference reported in proximal tibia morphometry between Asian and Caucasian populations, and the currently available implants do not account for the anthropometric variations observed across ethnicities. We aimed to evaluate the proximal tibia anthropometry in a Pakistani population.

Materials and methods

A study was conducted at The Indus Hospital, Karachi Campus, from August 2019 to July 2020. All consecutive patients fulfilling the eligibility criteria and undergoing knee replacement surgery were included in the study. Baseline characteristics and anthropometry of proximal tibia were recorded on a pre-designed proforma. Statistical analysis was done using SPSS version 24.

Results

A total of 30 patients were enrolled in this study, which included 17 females (56.7%) and 13 males (43.3%). The mean age was 61.6± 7.9 years and the BMI was 33±5.7 kg/m2. There was a significant difference found in the anteroposterior and mediolateral dimensions in both genders. A significant association was noted with body mass index (p-value 0.01) and occupation (p-value=0.02).

Conclusion

The results indicated that the anatomical profile of the proximal tibia in the Pakistani population is distinct, thus stressing the fact that it requires developing prostheses specifically tailored to this population's sizing requirements.

## Introduction

Knee osteoarthritis (OA) is a common reason for pain and disability and is the most common form of arthritis [1,2]. The knee joint, which can be divided into three compartments: medial, lateral, and patellofemoral, is considered as a modified hinge joint. Usually, the lateral and medial articular surfaces of the femur and tibia are asymmetrical, with the distal surface of the medial femoral condyle being narrower and more curved than that of the lateral condyle. The lateral articular surface of the tibia is more circular in shape, while the medial surface is oval with a longer anteroposterior axis. The knee joint undergoes significant motion, bears the body weight and stresses, and hence is prone to OA. The overall prevalence of OA is about 13.9% in adults aged 25 years, rising to 33.6% in those 65 and older, affecting around 18.7% of women and 13.5% of men in this age group [3]. Effective treatment methods for OA knees (depending on the stage of the disease) include both operative and non-operative therapies, which are tailored to the patient's specific situations and characteristics.

Total knee arthroplasty (TKA) is the most common surgical intervention for advanced knee OA [2,4], with other surgical options such as high tibial osteotomy and uni-compartmental replacement being less common. Nonetheless, about 10% to 30% of post-TKA patients experience continued pain and disability [5], often due to a bone-implant mismatch. The long-term success of total TKA implants depends on optimal bone resection and maximum coverage of the resected bone by appropriately sized implants [6]. However, most of the TKA implants available commercially do not account for these anthropometric differences observed across different ethnicities, as indicated by previous studies [7,8]. Significant variations in knee morphology have been observed between the Asian and Western populations in the literature previously [8]. It has been observed that there is a difference in proximal tibia morphometry with respect to its anteroposterior (AP) and mediolateral (ML) dimensions and tibial slope in Asian and Western populations [9]. A study reported from Korea demonstrated the mean ML and middle AP dimensions to be around 73.5 ± 5.6 mm and 47.3 ± 3.8 mm, respectively with males having larger dimensions compared to females [7]. Similar results were noted in an Indian population where males had larger values for both the AP and ML dimensions compared to females (47.80 ± 3.65 mm vs 43.53 ± 3.40 mm and 72.02 ± 5.13 mm vs 67.58 ± 5.69 mm, respectively) [9].

However, the number of patients undergoing TKA is increasing in Pakistan, and after COVID-19, the availability of implants has seen a significant decrease, due to transportation and travel restrictions. Currently, no research data about proximal tibia anthropometry in our population is available which can guide us about the common sizes used in the population. All studies in the past have been either done radiologically or on dry bone models. Accurate information will help in choosing the right implant for TKA for a population of Pakistani origin. We aim to generate local data for the development of the total knee system according to our population-based anthropometry.

## Materials and methods

An observational cross-sectional study was conducted at the Department of Orthopedics at The Indus Hospital in Karachi, Pakistan. After the approval of the Institutional Review Board (IRB), all patients meeting the inclusion criteria, aged between 35 and 75 years old and undergoing TKA for OA knees, were included in the study. Patients with a history of malunited proximal tibia fracture, significant bone loss, with secondary arthritis, and those patients undergoing unilateral knee replacement/revision total knee replacement (TKR) were excluded from the study.

Bilateral TKR was performed by senior orthopedic surgeons with more than 10 years of experience and after the standard proximal tibia bony cut, the anteroposterior and mediolateral dimensions of the right and left knees were measured via Vernier calipers by the operative surgeon. The readings were taken two times and the average value was recorded. 

A pre-designed proforma was used to gather information such as age, gender, ethnicity, height, weight, BMI, marital status, pre-operative range of motion, pre-operative fixed flexion deformity, and varus-valgus deformity. Data was entered and analyzed using SPSS version 24.0. Mean ± SD or Median (IQR) was computed for all the quantitative variables like current age, height, weight, BMI, preoperative range of motion, and mediolateral and anteroposterior dimensions as appropriate.

All categorical variables, such as gender, ethnicity, marital status, occupation, under- or over-coverage of the tibial plateau, preoperative fixed flexion deformity, and varus-valgus deformity were analyzed by computing their frequencies and percentages. To minimize the impact of effect modifiers, the data was stratified based on various variables such as age, gender, ethnicity, occupation, marital status, BMI, pre-op fixed flexion deformity, and varus-valgus deformity. By doing so, the potential influence of these variables on the study outcomes was controlled, allowing for a more accurate analysis of the relationship between the variables of interest.

Post-stratification independent sample t-test, Mann-Whitney U test, one-way ANOVA, or Kruskal-Wallis test was applied as appropriate to assess significant differences in mediolateral and anteroposterior dimensions among the stratified variables; p-value<0.05 is considered statistically significant.

## Results

A total of 30 patients were enrolled in this study of which 17 were females (56.7%) and 13 were male patients (43.3%). The mean ± SD age was 61.6 ± 7.9 years and BMI was 33±5.7 kg/m^2^. Considering the range of dimensions (minimum and maximum values), the AP dimension on the medial side was 34-59 mm (right) and 35-60 mm (left), the AP dimension on the lateral side was 35-61 mm (right) and 34-57 mm (left), and the ML dimension was 62-82 mm (right) and 60-89 mm (left). The patient characteristics and co-morbidities are shown in Table 1.

**Table 1:** Characteristics of the study participants HTN: hypertension, DM: diabetes mellitus

Parameters	Values
Gender (n%)	
Male	13 (43.3)
Female	17 (56.7)
Total	30 (100)
Age (Years)	
Mean ± SD	61.6 ± 7.9
Min-max	46-75
Height (cm)	
Mean ± SD	155.3 ± 8.6
Min-max	140-177
Weight (kg)	
Mean ± SD	79.3 ± 13.1
Min-max	55-106
BMI (kg/m^2^)	
Mean ± SD	33 ± 5.7
Min-max	20.7-43.1
Comorbid (n%)	
HTN	20 (66.7)
DM	7 (23.3)
Other	3 (10)
Total	30 (100)
Ethnicity (n%)	
Punjabi	2 (6.7)
Sindhi	3 (10)
Urdu-speaking	22 (73.3)
Pathan	2 (6.7)
Other (Specify)	1(3.3)
Total	30(100)
Occupation (n%)	
Professional	3 (10)
Technician or associate professional	2 (6.7)
Service and sales workers	3 (10)
Plant or machine operator	2 (6.7)
Elementary occupations	2 (6.7)
Housewife	15 (50)
Retired‎/pensioner	3 (10)
Total	30 (100)
Marital status (n%)	
Married	26 (86.7)
Unmarried	1 (3.3)
Widow	3 (10)
Total	30 (100)
Pre-operative min range of motion right knee(Degree Â°)	
Min-max	0-35
Median (IQR)	2.5 (0-10)
Pre-operative max range of motion right knee (Degree Â°)	
Mean ± SD	106.5 ± 12.4
Min-max	90-130
Fixed flexion deformity Right knee (degree Â°)	
Min-max	0-35
Median (IQR)	2.5 (0-10)
Varus deformity right knee (degree Â°)	
Mean ± SD	4.8 ± 5.2
Min-max	0-15
Mediolateral dimension right knee (mm)	
Mean ± SD	71.4 ± 5.2
Min-max	62-82
Anteroposterior dimension medial right knee (mm)	
Mean ± SD	45.4 ± 6.3
Min-max	34-59
Anteroposterior dimension lateral right knee (mm)	
Min-max	35-61
Median(IQR)	43.5 (40-48)
Pre-operative min range of motion left (Degree Â°)	
Min-max	0-40
Median (IQR)	0 (0-10)
Pre-operative max range of motion left knee (Degree Â°)	
Min-Max	90-130
Median (IQR)	110(90-110)
Fixed flexion deformity left knee (degree Â°)	
Min-max	0-40
Median (IQR)	0 (0-10)
Varus deformity left knee (degree Â°)	
Min-max	0-15
Median(IQR)	0 (0-10)
Mediolateral dimension left knee (mm)	
Mean ± SD	71.6 ± 6.4
Min-max	60-89
Anteroposterior dimension medial side left knee (mm)	
Mean ± SD	45.6 ± 6.7
Min-max	35-60
Anteroposterior dimension lateral side left knee (mm)	
Mean ± SD	44.4 ± 6.8
Min-max	34-57

There was a significant gender-wise difference in BMI, anteroposterior dimension of the lateral side, and mediolateral dimension in both genders (Table 2). The mediolateral dimension apart from gender was significantly associated with employment status (Table 3).

**Table 2:** Gender-wise differences in patient characteristics ꝉ: Mann Whitney U test, µ: Independent sample t-test, *P-value<0.05,**P-value<0.0001

Parameters			
Gender	Male n=13	Female n=17	P value
Age in years			
Mean ± STD	64.3 ± 7.9	59.5 ± 7.5	0.097^µ^
Min-max	47-75	46-70	
BMI			
Mean ± STD	30.1 ± 5.4	35.3 ± 4.9	0.010^µ^
Min-max	20.7-38.2	25.6-43.1	
Pre-operative range of minimum motion right knee (Degree Â°)			
Min-max	0-35	0-30	0.300^ꝉ^
Median (IQR)	0 (0-7.5)	5 (0-10)	
Pre-operative range of maximum motion right knee (Degree Â°)			
Mean ± STD	108.5 ± 11.4	105 ± 13.2	0.458^µ^
Min-max	90-120	90-130	
Fixed flexion deformity right knee (degree Â°)			
Min-max	0-35	0-30	0.300^ꝉ^
Median (IQR)	0 (0-7.5)	5 (0-10)	
Varus deformity right knee (degree Â°)			
Mean ± SD	5.8 ± 5.7	4.1 ± 4.8	0.395^µ^
Min-max	0-15	0-10	
Mediolateral dimension right knee (mm)			
Min-max	62-82	64-78	0.006^*^^ꝉ^
Median (IQR)	75 (70.5-80)	69 (67.5-70)	
Anteroposterior dimension right knee medial side (mm)			
Mean ± SD	47.8 ± 7.8	43.6 ± 4.2	0.097^µ^
Min-max	35-59	34-52	
Anteroposterior dimension right knee lateral (mm)			
Min-max	35-56	36-61	0.020^*^^ꝉ^
Median (IQR)	48 (43-51.5)	42 (40-45)	
Preoperative range of minimum motion left knee (Degree Â°)			
Min-max	0-30	0-40	0.320^ꝉ^
Median (IQR)	0 (0-10)	0 (0-22.5)	
Preoperative range of maximum motion left knee (Degree Â°)			
Mean ± SD	109.2 ± 10.4	102.6 ± 12.8	0.141^µ^
Min-max	90-120	90-130	
Fixed flexion deformity left knee (degree Â°)			
Min-max	0-30	0-40	0.320^ꝉ^
Median (IQR)	0 (0-10)	0 (0-22.5)	
Varus deformity left knee (degree Â°)			
Min-max	0-15	0-15	0.183^ꝉ^
Median (IQR)	5 (0-10)	0 (0-6)	
Mediolateral dimension left knee (mm)			
Min-max	60-89	64-82	0.010^*^^ꝉ^
Median (IQR)	76 (70-78.5)	68 (65.5-70)	
Anteroposterior dimension left knee medial side (mm)			
Mean ± SD	48.1 ± 7.8	43.6 ± 5.3	0.074^µ^
Min-max	35-60	36-57	
Anteroposterior dimension left knee lateral side (mm)			
Min-max	36-56	34-57	0.007^*^^ꝉ^
Median (IQR)	48 (43-55)	41 (39-44.5)	

**Table 3:** Side-wise differences in mediolateral dimension between age groups, gender, BMI, and employment status ꝉ Mann Whitney U test, µ Independent sample t-test,*P-value<0.05,**P-value<0.0001

Parameter	n	Mediolateral dimension Right Knee (mm)			Mediolateral dimension Left Knee (mm)		
		Mean ± SD/ Median (IQR)	Min-Max	P value	Mean ± SD/ Median (IQR)	Min-Max	P value
Age (years)							
<63	14	71.8 ± 5.5	62-82	0.686^µ^	71.7 ± 7.6	60-89	0.950^µ^
≥63	16	71 ± 5.1	64-80		71.6 ± 5.4	64-82	
Gender (n)							
Male	13	75(70.5-80)	62-82	0.006^*^^ꝉ^	76(70-78.5)	60-89	0.010^*^^ꝉ^
Female	17	69(67.5-70)	64-78		68(65.5-70)	64-82	
BMI (kg/m^2^)							
<33.78	14	72.1 ± 4.2	67-80	0.453^µ^	72.5 ± 4.6	66-82	0.497^µ^
≥33.78	16	70.7 ± 6	62-82		70.9 ± 7.7	60-89	
Employment (n)							
Employed	12	74.3 ± 6.1	62-82	0.021^*^^µ^	76(68.5-78.8)	60-89	0.072^ꝉ^
Unemployed	18	69.4 ± 3.4	64-78		70(66.8-70.5)	64-82	

Furthermore, no significant difference was seen in both anteroposterior-medial sides (right and left); differences in the anteroposterior-medial dimensions in the right knee showed a p-value of 0.758, and anteroposterior-medial dimensions in the left knee showed a p-value of 0.955 between age groups (Table 4). 

**Table 4:** Side-wise differences in the anteroposterior medial dimension between age groups, gender, BMI, and employment status ꝉ:Mann Whitney U test, µ:Independent sample t-test,*P-value<0.05,**P-value<0.0001

Parameter	n	Anteroposterior dimension Medial side Right knee (mm)			Anteroposterior dimension Medial side Left knee (mm)		
		Mean ± SD/ Median (IQR)	Min-max	P value	Mean ± SD/ Median (IQR)	Min-max	P value
Age (years)							
<63	14	45.8 ± 5.6	39-59	0.758^µ^	45.6 ± 7.1	36-60	0.955^µ^
≥63	16	45.1 ± 7	34-58		45.5 ± 6.7	35-60	
Gender (n)							
Male	13	47.8 ± 7.8	35-58	0.097^µ^	48.1 ± 7.8	35-60	0.074^µ^
Female	17	43.6 ± 4.2	34-59		43.6 ± 5.3	36-57	
BMI (kg/m^2^)							
<33.78	14	44.9 ± 6	6-35	0.665^µ^	45.2 ± 6.3	35-60	0.794^µ^
≥33.78	16	45.9 ± 6.7	6.7-34		45.9 ± 7.3	36-60	
Employment (n)							
Employed	12	47.7 ± 7	35-59	0.107^µ^	47.5(41.3-52.3)	38-60	0.121^ꝉ^
Unemployed	18	43.9 ± 5.4	34-57		43(40-48.5)	35-57	

There was a significant difference in both sides (right and left) at the anteroposterior-lateral with a p-value of 0.02 on the anteroposterior-lateral right and 0.007 on the anteroposterior-lateral left dimensions between both genders. However, no significant differences were observed on the AP-medial right (p=0.822) and AP-medial left (p=0.953) dimensions between age groups (Table 5).

**Table 5:** Side-wise differences in the anteroposterior lateral dimension between age groups, gender, BMI, and employment status ꝉ:Mann Whitney U test, µ:Independent sample t-test,*P-value<0.05,**P-value<0.0001

Parameter	n	Anteroposterior dimension Lateral side Right knee(mm)			Anteroposterior dimension Lateral side left knee(mm)		
		Mean ± SD/ Median (IQR)	Min-Max	P value	Mean ± SD/ Median (IQR)	Min-Max	P value
Age (years)							
<63	14	42.5(40-48)	38-61	0.822^µ^	44.3 ± 7.2	34-57	0.953^µ^
≥63	16	45(40-49.3)	35-56		44.4 ± 6.6	34-56	
Gender (n)							
Male	13	48(43-51.5)	35-56	0.020^*^^ꝉ^	48(43-55)	36-56	0.007^*^^ꝉ^
Female	17	42(40-45)	36-61		41(39-44.5)	34-57	
BMI (kg/m^2^)							
<33.78	14	44.2 ± 5.5	35-56	0.630^µ^	44.2 ± 6.2	35-56	0.911^µ^
≥33.78	16	45.3 ± 6.7	36-61		44.5 ± 7.5	34-57	
Employment (n)							
Employed	12	47.5(46-50.8)	40-56	0.002^*^^ꝉ^	47(42.5-54)	34-56	0.060^*^^ꝉ^
Unemployed	18	41(40-43.8)	35-61		40.5(39-45)	34-57	

## Discussion

Obtaining accurate anthropometric data is crucial, as poorly shaped and sized prostheses can lead to significant soft tissue impingement. Literature has shown that there are differences in knee morphology between males and females [10-12], with females having a smaller dimension. Similar findings were noted in our study where a significant difference was noted in gender. However, no significant difference was observed between the right and left sides of the same individual.

However, ethnic differences are not taken into account, and most TKA implants manufactured in Western countries are designed for the Caucasian population's knee morphology. Implants designed for Caucasian patients have anatomical differences compared to Asian patients along with different tibia torsion angles and alignment which needs to be considered [11]. Although different ethnicity within the same population was not considered in our study, however, employment status had a significant association with the dimensions of the proximal tibia, giving the impression that excessive workload can alter the dimensions. A unique finding in our study was that body mass index had a significant association with the dimensions of proximal tibia.

While the femoral component maintains a balanced flexion-extension gap in TKA with the mediolateral part of the femoral component maintaining patellofemoral tracking, the tibia baseplate covering the resected surface as much as possible is of equal importance. Overhanging or undersized tibia components lead to soft tissue irritation and subsidence of the component in the long term [12].

Previous studies have shown that tibial components tend to contribute more towards complications [10], possibly because of a mismatch between the component and the proximal tibia. This mismatch is probably due to the dimensions of the proximal tibia. Asymmetry of the proximal tibia with a wider medial plateau and lateral plateau being longer is reported in studies [11].

Most of the TKA implants available in Pakistan are from Western countries and discrepancies between the anatomical features of Asian and Caucasian knees are not taken into consideration. One reason might be the lack of data available and secondarily due to ease of supply. A study by Yue et al [12] reported that even in developed countries like China, a majority of TKA systems used cause ML overhang of the component. Therefore, consideration of the target population's morphology and dimensions becomes immensely important [13, 14].

A study by Mahfouz et al. [15] on the 3D analysis of knee morphology reported that African American males and females had bigger AP dimensions than their Asian and Caucasian counterparts, while Asian males and females have smaller AP dimensions than the Caucasian population.

In another study by K.C. Lakati et al. [16], in adult Kenyan populations, proximal tibial anthropometry showed no significant difference in dimensions between the right and left sides similar to our study, but the medial condyle was longer and wider than the lateral condyle, with a longer anteroposterior dimension by an average of 4.5 mm and a wider mediolateral width by an average of 1.2 mm. Similar findings were reported in other studies as well in other populations and are also comparable to our report [13,17]. A published study from Korea in 2015 also had similar findings to our study with comparable ML and AP dimensions to our study population. This study also reported that males had significantly higher values for AP and ML dimensions than females [13]. A study by Bansal et al. from India has also reported similar findings [18].

It is important not only to be aware of the possibility of anatomical variation among population, gender, and ethnicity, but also among those of different employment classes. Although there are several limitations in our study, including a small sample size and comparison of the actual measures size and size of implant used and a long-term outcome, however, we find that this study can serve as a benchmark for further studies in this aspect which can ultimately lead to better outcomes of TKA.

## Conclusions

Our study is the first report of differences in dimensions among different genders and ethnicities in the Pakistani population and provides a guide through which manufacturers can provide better-sized and shaped implants to the surgeon which could lead to better outcomes with better-fitted implants. However, considering the large number of TKA taking place at different centers throughout the country, there is a need to further evaluate this aspect with larger-scale studies for the benefit of patients.
